# Risk of venous thromboembolism during rehabilitation of patients with spinal cord injury

**DOI:** 10.1371/journal.pone.0193735

**Published:** 2018-03-28

**Authors:** Sabine Eichinger, Lisbeth Eischer, Hana Sinkovec, Gabriela Wittgruber, Ludwig Traby, Michael Kammer, Paul A. Kyrle, Oskar Steinbrecher, Herbert Kaloud, Victoria Kyrle, Hartwig Moser, Renate Wildburger

**Affiliations:** 1 Dept. of Medicine I, Medical University of Vienna, Vienna, Austria; 2 Karl Landsteiner Institute of Clinical Thrombosis Research, Vienna, Austria; 3 Center for Medical Statistics, Informatics and Intelligent Systems, Medical University of Vienna, Vienna, Austria; 4 Allgemeine Unfallversicherungsanstalt Rehabilitation Clinic Tobelbad, Tobelbad, Austria; Medical University Innsbruck, AUSTRIA

## Abstract

**Background:**

Patients with spinal cord injury (SCI) are at risk of thrombosis and bleeding. Data on the risks during rehabilitation are inconsistent, and thromboprophylactic strategies are heterogeneous. We aimed to evaluate the thrombotic risk and bleeding events of SCI patients during rehabilitation.

**Methods:**

We retrospectively collected hospital record data of 263 consecutive SCI patients admitted at a rehabilitation clinic. 78 patients with acute venous thromboembolism (VTE) at the primary center, without acute trauma or lower extremity paresis, less than one month rehabilitation, or reasons for long-term therapeutic anticoagulation, were excluded. All patients received pharmacologic thromboprophylaxis throughout rehabilitation. Primary endpoint was objectively diagnosed VTE; secondary endpoint was bleeding.

**Results:**

Of 185 patients, 162 (88%) were men; mean age was 47.8 years. 94 patients were tetraplegic, 91 paraplegic. During a mean (±SD) time of 5.1±2.1 months, VTE was diagnosed in 8 patients. After excluding five patients with VTE detected within 2 days after admission, the probability of developing VTE after 6 months of rehabilitation was 2% (95% CI 0–4.4%). Only high D-Dimer upon admission was associated with risk of VTE (adjusted HR 2.3, 95% CI 1.4–4.1). Of 24 bleedings, 14 (64%) occurred at the heparin injection site. Two patients had major bleeding and five had clinically relevant non major bleeding.

**Conclusion:**

SCI patients are at risk of VTE and bleeding during rehabilitation. Strategies need to be developed to identify these patients in order to initiate adequate anticoagulation. Direct oral anticoagulants, which have a favourable risk-benefit profile and are convenient, should be explored.

## Introduction

Patients with major trauma, particularly those with spinal cord injury (SCI), are at high risk of thrombosis [1]. Without thromboprophylaxis up to 90% of patients develop a venous thrombotic event during the acute phase [1–3]. While a decline of the thrombotic risk over time is noted [3,4], data on the actual risk during the subacute phase are inconsistent. In some studies, an incidence of venous thromboembolism [VTE] in patients with SCI during the rehabilitation phase as high as 6 to 10% has been reported [5–7]. Conversely, in a Californian discharge database, the risks of VTE of SCI patients in the first three months, at six months, and at one year after injury were 34%, 1.1%, and 0.4%, respectively [8]. In a systematic review of seven studies, incidences of pulmonary embolism and deep vein thrombosis in the sub-acute phase after SCI, i.e. between three and six months after the trauma, ranged from 0.5% to 6.0% and from 2.0% to 8.0%, respectively [9]. All these studies were heterogeneous in populations, design and outcome reporting.

Older age has been identified as one of the risk factors of VTE after SCI [10,11]. Other risk factors apparently associated with increased rates of VTE include paraplegia (versus tetraplegia), motor complete (versus incomplete) injuries, concomitant lower-extremity fractures and presence of comorbidities [3,10,11]. The relevance of additional risk factors including sex or obesity is disputed. Most of these risk factors have been established during the acute phase, and data on thrombotic risk factors during the rehabilitation phase are sparse.

In patients with SCI, pulmonary embolism is a leading cause of death. The National Spinal Cord Injury Statistical Center Mortality reports pulmonary embolism in 3.1% of patients as the primary cause of death [12]. Thromboprophylactic regimens consisting of (low molecular weight) heparin and/or mechanical compression are implemented near universally in the acute care facilities [9,13]. Few studies address the optimal duration of prophylaxis for patients with SCI [14–16]. Pending firm evidence, it is nonetheless commonly agreed that three months is a reasonable time for thromboprophylaxis in most patients [10,12]. Thromboprophylactic strategies beyond three months are heterogeneous and far from being standardized. This is mainly due to insufficient data on the actual risk of VTE and a definition of risk factors. To complicate matters, patients in the rehabilitation phase have an increased bleeding risk because of frequent invasive interventions and falls.

Using a cohort of well-characterized SCI patients admitted to a large rehabilitation clinic we aimed to evaluate the long-term risk of VTE and thrombotic risk factors in patients with acute SCI during rehabilitation care. In addition, we collected data on bleeding events.

## Materials and methods

### Patients and design

We retrospectively collected hospital record data of 263 consecutive patients with SCI who had been admitted at the Allgemeine Unfallversicherungsanstalt Rehabilitation Clinic Tobelbad in Austria between January 2007 and February 2017. The study was approved by the Ethics Committee of the Allgemeine Unfallversicherungsanstalt. Obtaining patients’ informed consent was waived because all data were fully anonymized before they were accessed by any of the authors.

Patients younger than 15 years (n = 1), with an acute VTE at the primary care center (n = 12), admitted for other reasons than acute trauma (n = 28), without lower extremity paresis (n = 3), with late admission (> 3 months) after the acute trauma (n = 20), with less than 1 month rehabilitation (n = 8), or with other reasons for long-term anticoagulation at therapeutic dose (n = 6), were excluded.

All patients were transferred from outside primary care centers to the rehabilitation clinic, entered the study upon admission and were followed until discharge. Data of all eligible patients, including demographic information, SCI characteristics (location of spinal damage, type of residual neurological disability and spasticity), co-morbidities (e.g. diabetes, hypertension, cancer, cardiovascular disease, substance abuse), length of stay at primary care center, type and duration of thromboprophylaxis, time and type of invasive interventions, and date of discharge from the rehabilitation clinic were collected into a database. To define the extent and severity of SCI we used the American Spinal Injury Association Impairment Scale (AIS) [17]. All patients had low molecular weight heparin (LMWH) at prophylactic dose (dose range 4000–7500 IE once daily) and compression stockings throughout their stay at the rehabilitation clinic. LMWH was stopped at least 12 hours before surgical intervention.

The primary study endpoint was deep vein thrombosis and/or pulmonary embolism objectively verified by ultrasound, venography, computed tomography or ventilation/perfusion lung scanning, respectively, between admission to and discharge from the rehabilitation clinic. Diagnostic management was based on establishing the clinical probability of VTE followed by imaging techniques in case of high probability or high D-Dimer if probability was low. Upon admission, D-Dimer levels were routinely measured. In patients with high D-Dimer levels, bilateral compression ultrasound or venography depending on feasibility and/or a CT scan were performed. The secondary endpoint was the occurrence of a bleeding event. Major bleeding was defined according to the definition provided by the International Society on Thrombosis and Haemostasis [18].

D-Dimer levels were measured in plasma upon admission of the patients by quantitative immune assays.

### Statistical analyses

Patient baseline characteristics are described by mean and standard deviation, if the variable is continuous, and by absolute frequency and percentages, if the variable is categorical. The time to the first VTE during rehabilitation was analyzed with the use of time-to-event methods. Patients that were discharged from the rehabilitation clinic were considered as censored. To assess the association of VTE with age, sex and risk factors, univariable and multivariable Cox proportional-hazards models were used where age, sex and each risk factor were included as covariates. As the distribution of D-Dimer was skewed, the values were log-base-2-transformed before statistical analysis. Because only eight thrombotic events were observed and only one in a female patient, the effect estimates of standard Cox regression models are likely to be biased upward. To prevent this we applied the Firth’s penalized maximum likelihood bias reduction method for Cox regression [19]. The comparisons between groups of patients are therefore reported as cause-specific hazard ratios (HR) with ninety-five per cent profile penalized likelihood confidence intervals (CI), and *p*-values that refer to penalized likelihood ratio tests with the significance level of 0.05. The probability of VTE was estimated with the use of the Kaplan-Meier method. SAS V9.4 (2014, SAS Institute Inc., Cary, NC, USA) and R (Version 3.2.4, 2017, R Core Team, Vienna, Austria) were used for statistical analysis.

## Results

### Patients

The characteristics of 185 patients included in the final analysis are shown in Table 1. Mean age upon admission was 47.8 years and the majority was men (162, 88%). The average time interval between acute trauma and admission at the rehabilitation clinic was 6.5 ± 3.1 weeks. Ninety-four patients were tetraplegic and 91 paraplegic; injuries were motor-complete in 108 and incomplete in 77 patients. One patient had a VTE prior to the trauma. One patient died during rehabilitation from hepatic failure.

**Table 1:** Baseline characteristics of 185 patients with spinal cord injury (SCI).

Characteristic	Value
Age at admission, years	47.8 ± 18.3
Age >70 years, n (%)	26 (14.1)
Men, n (%)	162 (88)
Body mass index, kg/m^2^	24.6±3.5
Cause of SCI, n (%)	
Fall	121 (65)
Traffic accident	48 (26)
Blow	14 (8)
Bullet wound	2 (1)
Type of plegia, n (%)	
Tetraplegia	94 (51)
Paraplegia	91 (49)
AIS, n (%)	
A	59 (32)
B	10 (5)
C	39 (21)
D	77 (42)
VTE prior to SCI, n (%)	
Comorbidities	1 (0.5)
Cancer	2 (1)
Inflammatory bowel disease	1 (0.5)
Liver or renal disease	5 (2.7)
Death at rehabilitation clinic, n (%)	1 (0.5)
Time between trauma and admission, weeks	6.5 + 3.1
Time in rehabilitation clinic, months	5.1±2.1

± values are means ± SD

AIS… American Spinal Injury Association Impairment Scale VTE… venous thromboembolism

### Risk of venous thromboembolism

During a rehabilitation time of 5.1±2.1 months, 8 patients (7 men) were diagnosed with VTE (five deep vein thrombosis plus pulmonary embolism, two isolated pulmonary embolism, one isolated deep vein thrombosis). Table 2 shows detailed characteristics of these patients.

**Table 2:** Characteristics of eight patients with spinal cord injury (SCI) and venous thromboembolism (VTE).

Sex	Age (y)	Location of VTE	Symptoms of VTE	Time of diagnosis after admission (days)	Type of plegia	AIS	VTE prior to SCI	Comorbidities
Male	76	PE+DVT	no	1	Para	A	No	History of ischemic stroke
Male	63	PE	no	1	Tetra	C	No	Tongue cancer in complete remission
Male	39	PE+DVT	no	1	Para	B	No	None
Male	47	PE+DVT	no	2	Para	A	No	None
Male	28	PE	yes	2	Tetra	C	No	None
Male	40	PE+DVT	yes	22	Tetra	A	No	None
Male	30	PE+DVT	no	33	Para	A	No	None
Female	72	DVT	yes	132	Tetra	C	No	Myelodysplastic syndrome, diabetes mellitus, arterial hypertension

PE…pulmonary embolism DVT…deep vein thrombosis

AIS…. American Spinal Injury Association Impairment Scale

The cumulative probability of diagnosing VTE during rehabilitation after 6 months was 4.7% (95% CI 1.4–7.9%). Five of eight VTE were diagnosed within two days upon admission to the rehabilitation clinic. It is safe to say that these events already occurred at the primary care center. When we excluded these five patients from the analysis, the cumulative probability of developing VTE during rehabilitation was 2% (95% CI 0–4.4%).

### Risk factors of venous thromboembolism

In univariable Cox proportional hazards models, we evaluated the association of age, sex, body mass index, type of paresis, and high D-Dimer with risk of VTE. Only high D-Dimer measured upon admission was significantly associated with a risk of VTE (Table 3). The association remained significant after adjustment for age and sex (HR per doubling 2.33, 95% CI 1.36–4.07). All VTEs occurred in patients with motor-complete injuries of the lower extremities.

**Table 3:** Hazard ratios (HR) of risk factors for venous thromboembolism in 185 patients with spinal cord injury.

	Crude HR (95% CI) *p*-value	Adjusted HR* (95% CI) *p*-value
Age, per 10 years increase	1.04 (0.72, 1.53) p = 0.87	1.04 (0.72, 1.53) P = 0.83
Men vs. women	0.75 (0.16, 7.15) p = 0.75	0.75 (0.17, 7.16) p = 0.77
BMI, per 5 kg/m^2^ increase	1.18 (0.43, 3.03) P = 0.11	1.17 (0.41, 3.04) P = 0.76
D-Dimer per doubling	2.38 (1.39, 4.09) P = 0.002	2.33 (1.36, 4.07) P = 0.003
Type of plegia,Tetra vs. para	0.91 (0.24, 3.53 p = 0.89	0.87 (0.21, 3.59) p = 0.86

* for age and sex

### Risk of bleeding

Twenty-three bleeding events were recorded in 21 patients (two patients had more than one event). Fourteen patients (64%) had haematomas at the LMWH injection site. Characteristics of seven patients (30%) with bleeding complications at other sites or major bleeding (one gastrointestinal, one intrathoracic) are shown in Table 4.

**Table 4:** Characteristics of seven patients with spinal cord injury and bleedings other than at the low molecular weight heparin injection site.

Sex	Age (years)	Location of bleeding	Days after admission	Type of plegia	AIS	Comorbidities
Male	47	Intrathoracic	1	Paraplegia	A	None
Female	72	Gastrointestinal	4	Tetraplegia	C	Myelodysplastic syndrome, diabetes mellitus, arterial hypertension
		Macrohematuria	15			
Male	66	Macrohematuria	1	Tetraplegia	A	None
Female	61	Hemorrhagic blister due to decubitus	1	Paraplegia	A	Diabetes mellitus
Female	66	Tracheal	7	Tetraplegia	A	Diabetes mellitus
Female	41	Macrohematuria	14	Paraplegia	A	None
Male	29	Hemorrhoidal	273	Tetraplegia	A	Aspirin for aortal dissection

AIS…. American Spinal Injury Association Impairment Scale VTE….venous thromboembolism

## Discussion

In patients with SCI, the risks of VTE and bleeding during the time of rehabilitation are insufficiently studied. As a consequence, thromboprophylactic strategies are heterogeneous and lack consensus. In our study of 185 SCI patients at a large rehabilitation clinic, eight patients (4,3%) were diagnosed with a deep vein thrombosis and/or pulmonary embolism, and 21 patients had bleeding events during average admission time of 5.1 months. Our study and findings have some distinct features that set them apart from other studies on the thrombotic risk in subacute SCI patients. In a cross-sectional study, five of 63 patients (7.9%) with SCI had a deep vein thrombosis between four and 42 months post-injury [20]. Chung and colleagues reported a more than two-fold higher rate of VTE in Chinese SCI patients compared to a matched healthy control group over a period of 12 years [21]. Giorgi-Pierfranceschi and colleagues prospectively followed 94 SCI patients and recorded the majority of VTE events during the first three months after SCI (20 out of 22 events– 90.9%), for an incidence of 34.4 events/100 patient-years in the first three months as compared to 0.3 events/100 patient-years thereafter [4]. In contrast to these studies, our focus was exclusively on evaluating the thrombotic (and bleeding) risk in a large number of SCI patients during the time of rehabilitation, i.e. a time when decisions on thromboprophylaxis are critical but often remain non-consensual.

All thrombotic events were diagnosed within 4.5 months after admission. In a retrospective study of 16 000 patients discharged from California hospitals, 90% of all thromboembolic events reported within one year after injury occurred in the first 91 days [3]. These observations reflect the notion that after an initial excessive risk, the incidence of VTE subsides. This is particularly striking, as many strong thrombotic risk factors–foremost immobilization—are persistent, and a ready explanation for the decline in the thrombotic risk needs yet to be provided.

Notably, five of the eight thrombotic events in our study were detected within two days after admission. It is safe to assume that these VTEs already occurred at the primary care center, and that the probability of developing VTE during the chronic rehabilitation phase is low provided that adequate thromboprophylaxis is applied.

That the diagnosis had been missed in several patients, must not be viewed as unexpected since patients with acute SCI may have attenuated or absent typical signs and symptoms of VTE, including pain, swelling or dyspnoea [16, 22]. At the start of rehabilitation, patients are probably more likely to present with classical VTE symptoms as they may have regained some motor-neurologic function and have been weaned from heavy posttraumatic pain medication. Nonetheless, our observation should stimulate discussions about the relevance of VTE screening in SCI patients. Missing a VTE diagnosis also means that patients are left untreated for a potentially fatal disease. Despite a relatively high diagnostic yield of deep vein thrombosis, routine performance of compression ultrasound is usually not recommended because of high costs and an uncertain clinical relevance of asymptomatic VTE in general. Use of D-Dimer as a VTE screening tool is also been often dismissed as all patients with recent SCI will have elevated levels resulting in an increase of imaging tests with the potential consequence of overdiagnosis and overtreatment. In our study, in four of five VTE cases detected upon admission, the diagnosis was established because of an elevated D-Dimer level. The high incidence of VTE diagnosed early after admission in our study should raise awareness among rehabilitation care providers about the thrombotic risk of SCI patients and a potentially atypical clinical presentation. Upon the slightest suspicion, appropriate measures—foremost imaging techniques—should follow to objectify the diagnosis. As regards D-Dimer, levels were routinely measured upon admission. Levels in all five patients diagnosed with VTE upon admission were excessively high, ranging between 3400 and 14000 ng/ml, and thus well above the usual diagnostic threshold of 500 ng/ml. Increasing the cut-off level of D-Dimer increases its diagnostic specificity, which may warrant further investigation also in post-acute SCI patients.

The relatively high frequency of patients with VTE diagnosed upon admission also warrants questioning about prior thromboprophylaxis. However, primary patient care took place in separate health care facilities outside the rehabilitation clinic. We therefore cannot comment on thromboprophylactic regimens before admission to the rehabilitation clinic.

In our study, only elevated D-Dimer upon admission was associated with the risk of VTE. The majority of our patients was men which is in line with findings in other studies [4,20] and likely explains that only one of eight thrombosis patients was female. None of the thrombosis patients had a motor-incomplete injury or a history of VTE prior to the trauma, which renders risk calculations for these factors meaningless.

The risk of bleeding is another major concern in SCI patients. Likewise as the thrombotic risk, the risk of bleeding is substantial during the acute phase and declines thereafter [23]. Nonetheless, particular attention needs to be paid during rehabilitation, as SCI patients are repeatedly exposed to specific situations with increased bleeding, in particular invasive interventions, urogenital complications, infections and falls. In our study, two patients had a major bleeding and eight had clinically relevant bleedings. In contrast, no major bleedings in SCI patients given LMWH thromboprophylaxis were reported in a prospective Italian study and data on other forms of bleeding are not provided [4]. All our study patients received LMWH thromboprophylaxis at the time of bleeding. We do not have sufficient information to comment on the management of these patients regarding thromboprophylaxis after the bleeding. Usually, antithrombotic drugs are stopped in bleeding patients which may then lead to a breakthrough of the underlying thrombotic risk. This underlines the importance of thromboprophylactic strategies based on balancing the risks of thrombosis and bleeding in this particular patient population.

Additional strengths and limitations of our study shall be addressed. We evaluated both the frequency of VTE and bleeding in a large cohort of patients admitted at a single rehabilitation center. Thus, the management of patients as regards diagnosis and treatment was uniform which adds to the homogenous nature of our study population. We applied adequate statistical analysis to assess the association of VTE with potential risk factors, but due to the low event rate our study may lack power to detect significant effects. The exclusion of patients with VTE or major bleeding at the primary care center may have influenced the event rates, which to some extent precludes comparison to other studies with different patient selection criteria. Our study design enables us to provide risks of thrombosis and bleeding relevant exclusively for the rehabilitation phase. As all patients received LMWH thromboprophylaxis, we have no information on the thrombotic risk in the absence of pharmacologic thromboprophylaxis.

In patients with SCI, thromboprophylaxis for a prolonged period is recommended in guidelines [10]. In our study, patients received prophylactic LMWH throughout their clinical stay, i.e. 5.1 months in average. In some other parts of the world, also UFH or warfarin is used instead of LMWH. All these drugs come with substantial inconvenience for the patients as they require daily injections, frequent laboratory monitoring or pose a risk of heparin induced thrombocytopenia. Of note, two thirds of bleeding events recorded in our study occurred at the LMWH injection site. Direct oral anticoagulants are effective and safe for primary and secondary prevention of thrombosis in patients with atrial fibrillation or VTE [24,25]. They are licensed for thromboprophylaxis at a reduced dose, but only for patients after hip or knee replacement where they are at least as effective and as safe as LMWH [26]. Only limited data on direct oral anticoagulants for thromboprophylaxis outside major orthopaedic surgery are available [27,28]. Because direct oral anticoagulants are given orally in a fixed dose without the need for laboratory monitoring, have very few drug and no food interactions, and are less costly than LMWH, they could be attractive compounds for thromboprophylaxis particularly during rehabilitation phase of SCI patients.

Patients with SCI are at risk of VTE during the rehabilitation phase. Strategies need to be developed to identify these patients in order to initiate adequate anticoagulation. In many institutions LMWH thromboprophylaxis is given beyond three months post-injury. At the same time, these patients are at increased risk of bleeding. Direct oral anticoagulants, which have a highly favourable risk-benefit profile and are convenient, should be explored in SCI patients during rehabilitation.
